# Using a final ecosystem goods and services approach to support policy analysis

**DOI:** 10.1002/ecs2.2382

**Published:** 2018-09-13

**Authors:** Paramita Sinha, Paul Ringold, George Van Houtven, Alan Krupnick

**Affiliations:** 1 RTI International, 701 13th Street, N.W., Suite 750, Washington, D.C. 20005 USA; 2 U.S. Environmental Protection Agency, 200 SW 35th Street, Corvallis, Oregon 97333 USA; 3 RTI International, 3040 East Cornwallis Road, Research Triangle Park, North Carolina 27709 USA; 4 Resources for the Future, 1616 P St. NW, Suite 600, Washington, D.C. 20036 USA

**Keywords:** atmospheric deposition, ecological valuation, ecosystem goods and services, final ecosystem goods and services, policy analysis, regional analysis

## Abstract

Evaluating environmental policies requires estimating the impacts of policy-induced changes on ecological and human systems. Drawing connections between biophysical and economic models is complex due to the multidisciplinary nature of the task and the lack of data. Further, time and resource constraints typically limit our ability to conduct original valuation studies to fit the specific policy context. Policy analysts thus rely on methods to transfer and adapt value estimates from existing studies. To conduct end-to-end policy analysis, assumptions are needed to make the linkages between ecological and valuation models as well as to conduct benefit transfers. This paper discusses an approach that can potentially help a policy analyst to minimize assumptions and identify appropriate caveats. This approach focuses on what human beings truly value from ecosystems, or, in other words, metrics of Final Ecosystem Goods and Services (FEGS). our hypothesis is that the FEGS approach will help support policy analysis by drawing important linkages between ecological and economic models as well as by designing valuation studies that will be more conducive to benefit transfers. To examine this hypothesis, we use a selected set of existing valuation studies as case study examples, and we examine how the methods used in these studies compare with the FEGS approach. We find that the studies are not always consistent with the FEGS approach, in many cases due to data limitations. We illustrate ways in which using FEGS metrics can provide economists with a useful starting point for considering how the commodity can be defined and specified in the valuation study. Even if data limitations exist, a FEGS approach can help in determining whether the context in which the original study was conducted matches with the policy context. This can also help in determining the extent of uncertainty associated with the analysis and in providing transparent documentation that can be informative for policy makers.

## Introduction

Environmental policy formulation requires information not only on how policy changes lead to biophysical changes in the environment, but also on how these biophysical changes link to impacts on human well-being. Conducting such an end-to-end policy analysis can therefore be broadly envisioned as a two-part process. The first part entails estimating policy-induced changes in the observable biophysical ecological attributes that people care about in ecosystems. (Natural scientists often use the term “attributes,” while economists refer to anything that human beings value as “commodities.” In this paper—meant for both audiences—we use both terms interchangeably.) The second part entails estimating the welfare impacts of these changes, which requires estimating the value that people attach to these attributes. Since ecological attributes are typically not traded in markets and do not have observable prices, estimating their implicit prices is often done using a variety of non-market valuation techniques. These valuation studies seek to quantify the value of changes in human welfare arising from changes in attributes of ecological resources and the associated ecosystem services. They provide monetary estimates of what people would be willing to pay for the ecological change.

Two main challenges associated with conducting end-to-end policy analysis are as follows. First, policy analysis requires integrating ecological and economic valuation models. Existing connections between ecological and valuation models are limited and drawing such connections is complex due to the interdisciplinary nature of the task and lack of data. Second, valuation studies designed specifically for the policy context in question are typically hard to find. Time and resources to conduct new valuation studies that provide context-specific estimates are not readily available. Consequently, analysts often conduct benefit transfers to assess welfare impacts of policies. Benefit transfer is the “practice of adapting value estimates from past research to assess the value of a similar, but separate, change in a different resource” (Smith et al. 2002). Accurate and credible benefit transfer thus depends on the contextual similarity of the original study and the policy in question. Too conduct end-to-end policy analysis, assumptions are needed to make the linkages between ecological and valuation models as well as to conduct benefit transfers. To conduct a successful policy assessment, it is important to minimize such assumptions as much as feasible. Where assumptions are unavoidable, transparency about the assumptions and associated caveats are informative to decision makers. This paper discusses an approach that can potentially help a policy analyst to minimize assumptions and identify associated caveats as appropriate.

A recently developed approach for identifying the linkages between ecosystems and human welfare applies the concepts of end-products from nature and measures of Final Ecosystem Goods and Services (FEGS; Boyd and Banzhaf 2007, Landers and Nahlik 2013, Ringold et al. 2009, 2011, 2013). End-products are “the components of nature, directly enjoyed, consumed or used to yield human well-being” (Boyd and Banzhaf 2007). The concept of end-products can be used to derive FEGS metrics, which directly contribute to human activities or well-being. Ecosystems also provide intermediate goods or services, such as fish habitat or nutrient processing, which are important to humans but considered intermediate because people benefit indirectly from them. (Note that while goods and services are technically quite different, we follow the common practice in the ecosystem literature and use services as a shorthand for goods and services.) For example, although recreational anglers benefit from nutrient cycling because it plays an essential role in supporting the growth and development of fish populations, it is the resulting stock of fish (the end-product) that matters most to them. As noted in Boyd and Banzhaf (2007), the values of intermediate ecosystem services are embodied in the value of the end-products they produce. In our example, the value of nutrient processing to anglers is embedded in the anglers’ value for the available fish stock. Given this distinction between intermediate and final services, a crucial step for conducting analysis of policy-induced changes in ecosystems on human welfare is to identify the relevant affected end-products and associated FEGS metrics, which represent the points of handoff from the ecosystem to human beings. More explicitly, this step involves identifying the ecosystem that provides the end-products and associated FEGS metrics as well as the beneficiaries who directly enjoy, consume, or use them (Landers and Nahlik 2013).

Although much of the work done to develop the FEGS concept has focused on ecological monitoring (Boyd and Banzhaf 2007, Ringold et al. 2009, 2011, 2013, Landers and Nahlik 2013), the concept can also be used to define an approach for conducting end-to-end policy analyses. In this paper, we define and refer to this as the “FEGS approach” for policy analysis. Our hypothesis is that because the FEGS approach focuses on what human beings truly value from ecosystems, it will help support policy analysis by drawing important linkages between ecological and economic models as well as by designing valuation studies that will be more conducive to benefit transfers. To examine this hypothesis, we use a selected set of existing valuation studies as case study examples, and we examine how the methods used in these studies compare with the FEGS approach.

The main goals of this paper are therefore to address the following questions. First, in what ways are the ecological attributes defined and valued in these case studies consistent or inconsistent with a FEGS approach? Second, where inconsistencies are found, do they create barriers for applying these studies to conduct end-to-end policy analysis? Third, in what ways could a valuation study be designed to use a FEGS approach so that these barriers can be reduced? In other words, could a FEGS approach help in designing valuation studies that allow for credible and transparent benefit transfers? It is important to note that this paper is not intended to be a critique of these particular studies because they were not necessarily designed for end-to-end policy analysis. Rather, the selected studies are used to highlight the types of barriers and limitations that an analyst faces when applying the value estimates to conduct end-to-end policy analysis.

In *Policy Framework and the FEGS Approach,* we describe a framework highlighting the key components for conducting an end-to-end policy analysis and defining the linkages between them. With this framework in hand, we define the FEGS approach and describe what is entailed in applying the FEGS approach to model welfare impacts of changes in ecological commodities. To examine and demonstrate this point, we translate the key features of the FEGS approach into two criteria, which we then use to evaluate the methods applied and commodities valued in a set of case studies.

In *Review of Valuation Methods and Selected Studies,* we describe the valuation studies selected as case studies for this paper. To narrow our scope, we define reductions in air pollutant deposition as our policy focus, and we select studies that primarily value regional changes in surface water quality, where the sources of impairment include atmospheric deposition of nitrogen and sulfur. Therefore, the water quality impairments addressed in these studies include acidity, eutrophication, toxicity, or a combination of these, and their associated impacts on aquatic ecosystems. (We note that policies lowering deposition of nitrogen and sulfur may also have associated co-benefits, such as reductions in toxicity, and these co-benefits are also considered in this paper.) We chose to focus on regional (rather than waterbody-specific) valuation studies and atmospheric deposition in part to allow for more explicit consideration of temporal and spatial variation of ecological impacts. We also selected the set of valuation studies to ensure that a mix of different valuation methods is represented.

In *Analysis of Case Studies*, we compare the methods used in these valuation studies using the FEGS approach criteria. Specifically, to address our first goal, we evaluate whether the ecological commodities valued in the studies satisfy the criteria developed in *Policy Framework and the FEGS Approach*.

In *Implications for Policy Analysis*, we focus on addressing our second and third goals. We examine the associated implications for end-to-end policy analysis when the commodities valued are not fully consistent with the FEGS approach. We conclude the paper with a discussion of key elements of the paper and recommendations for natural scientists and social scientists and suggestions for future research.

## Policy Framework and the Fegs Approach

### Framework for policy analysis

**Fig. 1.**
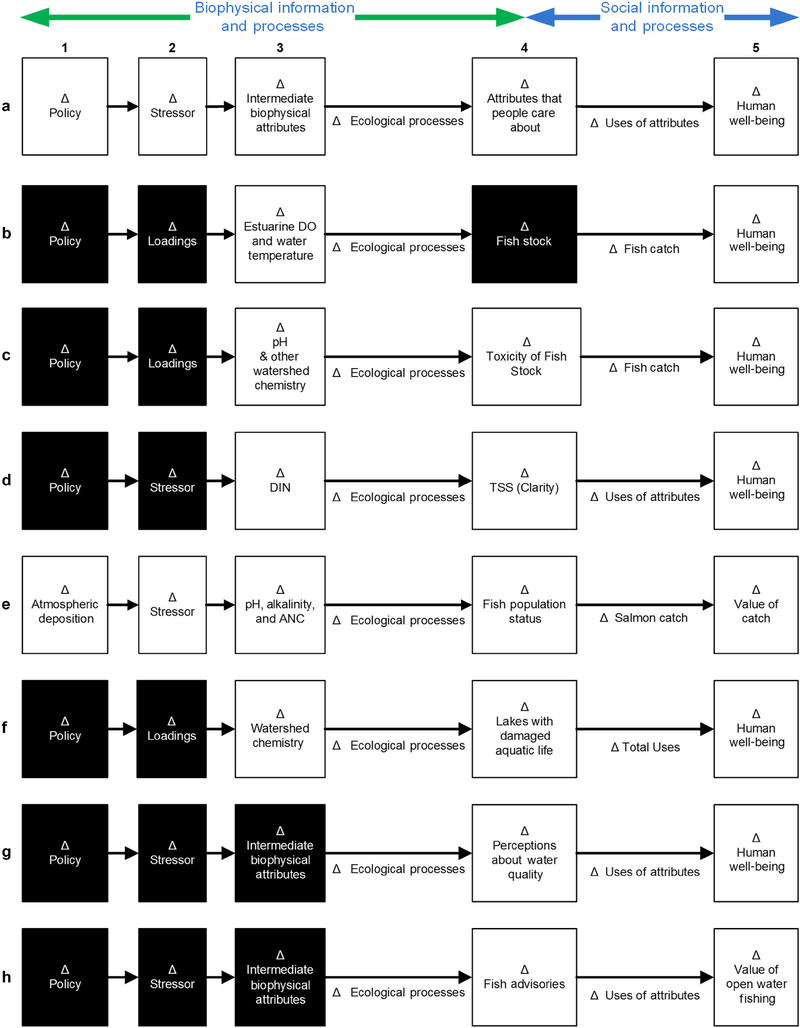
Policy analysis framework and case study illustration: (a) a general framework for end-to-end policy analysis; and (b–h) the manner in which each case study (listed in Table 1) fits into this framework. Components necessary to do policy analysis that are not included in the studies are blacked out.

Estimating the benefits resulting from policy changes requires clear cause-and-effect quantitative linkages between ecosystems and human well-being. Fig. 1a provides a framework that shows the pathway through which a change in policy translates into changes in valued attributes of ecosystems and consequently to impacts on human welfare.

The sequence of changes begins with a policy change (Step 1 in Fig. 1a) such as a more stringent secondary National Ambient Air Quality Standards for nitrogen oxides (NO_x_) and sulfur oxides (SO_x_), which would require reductions in emissions (under a situation where existing emissions lead to exceedances of the secondary standard). A change in policy leads to a change in the stressor (Step 2 in Fig. 1a) such as reduced atmospheric deposition of NO_x_/SO_x_. Estimates of changes in deposition may be derived from monitoring systems, for example, from the National Atmospheric Deposition Network, or from modeled estimates, such as those derived from applications of the Community Multiscale Air Quality modeling system. Changes in the stressor then cause changes in the biophysical attributes of ecosystems. When these attributes are not directly observed and valued by humans, we refer to them as intermediate attributes (Step 3 in Fig. 1a). For example, reduced deposition leads to changes in biogeochemistry within affected watersheds and to water quality changes in their aquatic ecosystems (e.g., pH levels in rivers and lakes). Changes to these attributes in turn impact ecosystem processes (e.g., fish reproduction), which result in changes to the ecological attributes or commodities (end-products) that people do care about (e.g., fish abundance; Step 4 in Fig. 1a). Changes in these end-products, which provide the FEGS that people directly benefit from, yield changes in human welfare (Step 5 in Fig. 1a).

The size and distribution of welfare changes depend on the level of change in the ecological attributes and the ways in which humans derive value from the affected ecosystems. People derive benefits from FEGS through the different ways they use or appreciate them. Thus, changes in benefits can potentially be derived from switching to higher valued uses of attributes or through receiving more value from same use. Both possibilities are represented by Δ Use in the figure (arrow between Step 4 and Step 5). For example, consider a lake where people wade but do not swim since they do not perceive the water quality to be good enough. An improvement in the water quality may result in some people swimming. It may also result in an increase in the number of people wading in the lake or the same number of people deriving greater value from wading due to improved water quality.

Modeling the welfare impacts of changes in ecological commodities requires estimating the monetized value that people attach to them. Economists use a variety of methods and models (described in *Review of Valuation Methods and Selected Studies* and Table 1) to estimate these values; however, successful end-to-end policy analysis requires valuation studies that fit the policy context of interest. To conduct credible benefit transfers, the commodity being valued needs to be defined such that two types of linkages can be established. First, there is the linkage from a policy change (P) to a change in final attributes being valued (F), represented by the green arrow in Fig. 1a. Valuation approaches should ideally define the commodity F in a way that can be quantifiably linked back to the policy P. For example, if a valuation study uses qualitative descriptions or subjective rankings to define changes in ecological conditions (see Stated preference studies for examples), it can be challenging to define how a policy change would affect these types of attributes. It is typically easier to link policy changes to changes in measurable biophysical attributes. Second, there is the linkage from F to human preferences and well-being (V) represented by the blue arrow in Fig. 1a. It is important to be able to trace a path from a change in the ecological attribute being measured to something that is readily comprehensible and clearly provides benefits to humans. For example, although pH is a biophysical indicator of acidification, it is not an attribute that an average angler can comprehend or value. (Even if anglers understand that pH is a proxy for fish health and abundance, they may not know exactly what level of pH is suitable for healthy fishing stock and/or the magnitude of increase in fish stock due to improved pH levels.) Therefore, if the only information we can derive from biophysical models is how changes in acidification affect pH, the challenge will be to develop a valuation approach that links human preferences to this type of indicator.

The strength of an end-to-end analysis will ultimately depend on the strength of these two linkages. Analyses that rely more heavily on assumptions and translations to make these linkages will typically have a higher degree of uncertainty.

### Defining and applying a FEGS approach

The notion of identifying measures that link analysis of ecosystems to the analysis of human well-being (Boyd and Banzhaf 2007, Landers and Nahlik 2013, Ringold et al. 2013) has led to the identification of FEGS metrics and methods for defining them. In this approach, the definition of “end-products of nature” was made operational for each ecosystem type. Broadly speaking, this was done by identifying, for each ecosystem, (1) the sectors of society and the economy that directly benefit from these end-products and (2) the different ways in which they benefit from these end-products. A six-step process was developed to identify the points of handoff between nature and humans, and these steps are described in detail elsewhere (Ringold et al. 2009, 2011, 2013).

Ringold et al. (2009, 2011, 2013) describe two key features of a FEGS approach that supports successful ecological monitoring are summarized in the first column of Table 2. Since these features also have an important bearing on modeling welfare impacts, we use them to define and interpret what adopting a FEGS approach in valuation studies entails. Specifically, we use them to define the criteria necessary for commodities valued in the economics literature to be consistent with a FEGS approach. The second column of Table 2 summarizes the evaluation criteria for modeling welfare impacts. We also describe how these two criteria help determine whether estimates can be easily and accurately applied to conduct end-to-end policy analysis.

To examine and illustrate the implications of these criteria for adopting a FEGS approach, we use seven existing environmental valuation studies as case study examples. We evaluate each case study by determining whether the commodity being valued satisfies the following two criteria.

*Criterion 1 (meets the definition of a FEGS metric): Ecological attributes being valued must be biophysical measurable entities and require little translation to make clear their relevance to beneficiaries.—*In short, this criterion defines the meaning of a FEGS metric. To determine whether the commodity being valued meets this definition, this criterion involves evaluating whether the commodity being valued (a) is a biophysical attribute that is a measurable entity (or can be explicitly tied to measurable entities), and (b) is an end-product and requires little translation to make clear their relevance to beneficiaries.

The specification of FEGS allows for both the P-to-F and F-to-V linkages to be established. If the commodity being valued is a biophysical measurable attribute (as opposed to qualitative descriptions or subjective rankings), then ecological models and data can be used to estimate the quantitative link between the policy and the commodity (P-to-F linkage). If the commodity being valued requires little translation to make clear their relevance to beneficiaries, then the F-to-V linkage is readily satisfied. No further translation or strong assumptions are required to conduct meaningful benefit transfers.

Further, our premise under the FEGS approach is that using end-products that are directly relevant to human beings provides more accurate estimates of value (relative to using intermediate metrics). This is due to several reasons. First, intermediate processes, functions, and services are embodied in the value of the final service. Second, some intermediate services may be difficult for people to relate to and meaningfully value directly (Boyd et al. 2016). Third, uncertainties may arise if people are asked to value intermediate goods and services since they implicitly translate them into the final goods and services that directly matter to them. This translation is based on their subjective understanding which, as non-experts, may introduce errors.

*Criterion 2 (involves defining complete set of ecosystem and beneficiary-specific ecological attributes including all biophysical, temporal, and spatial dimensions).—*Since people derive benefits from different sets of ecological attributes provided by ecosystems, all possible beneficiary-specific attributes must be represented in the valuation study. Temporal and spatial dimensions of the valued attributes must be well defined. To evaluate whether this second criterion is satisfied, we examine whether the attributes that are specifically relevant to the beneficiary (and to freshwater ecosystems) are included in the case study. People benefit from ecosystems in diverse ways, and each of these ways may require different representations of the ecosystem. For example, recreational fishermen may care about the stock of fish in lakes, while irrigators may care about the quantity of water in lakes available for irrigation. Consequently, the correct representation of ecosystem changes is important for obtaining accurate value estimates in the context of modeling welfare impacts. We also examine whether the valued commodity includes all possible ecological attributes that are likely to be relevant for a specific beneficiary. (We note that attributes whether natural or anthropogenic that provide or limit access to a resource of interest, for example, boat ramps, also consumed in conjunction with FEGS attributes. While considering such attributes is important for social analysis, following the description in Ringold et al. (2013) they are not the focus of this study nor are they considered as part of the FEGS.) Considering multiple attributes in benefits estimation is important since people typically benefit from a bundle of ecological attributes. Failure to include all relevant attributes may potentially result in inaccurate estimates. For example, recreational anglers benefit not only from the fish in a lake, but also from the aesthetics of the surroundings (Hunt 2005). These multiple attributes (or what economists call complements) are usually consumed together to yield benefits to a recreational fisherman. Thus, if a recreation valuation study includes only measures of fish abundance, but the policy of interest also improves aesthetics, then the full benefits of the policy for anglers would not be captured by the study.

To apply this second criterion, we draw heavily on Ringold et al. (2011, 2009) which identified the sets of attributes of freshwater ecosystems that are likely to be relevant to specific beneficiaries. We use these findings to obtain examples that we can compare to the commodities valued in the case studies. The underlying assumption is that these attributes have been correctly identified in these two studies. Table 3 provides a comparison of the beneficiary-specific attributes identified in these two studies (i.e., our benchmark) and the commodity valued in the case studies. The second column identifies the beneficiary that each case study focuses on. The third column provides examples of attributes relevant to these beneficiaries, as identified in Ringold et al. (2011, 2009). The last column lists the commodities valued in the case studies. When the authors do not focus on commodities that are consistent with a FEGS approach, we examine whether they provide a rationale for the commodity being used and what the rationale was.

Further, the values people hold for biophysical attributes are often location and time specific. For example, whereas flooding for a homeowner living next to a stream is always detrimental, flooding can be beneficial or detrimental for a farmer depending on the time of the flood. It can be detrimental if it occurs during the planting, growing, or harvesting season, or beneficial at other times of the year (Cuny 1991). Therefore, the relevant temporal and spatial dimensions of these attributes must be specified in as much detail as possible for accurate and transparent benefit transfers. To apply this criterion, we examine whether and how the case studies define the temporal and spatial characteristics of the valued commodity from specific ecosystem types.

## Review of Valuation Methods and Selected Studies

To examine and demonstrate these evaluation criteria and the FEGS approach with case study examples, we focus on a set of studies that can help to inform policy analysis of changes in atmospheric deposition and subsequent effects on aquatic ecosystems. This focus on atmospheric deposition was selected because it has been at the forefront of policy deliberations and therefore well analyzed from a biophysical and an economic perspective over the last several decades. Related explorations of the links between atmospheric deposition and ecosystem services using a FEGS framework can be found in O’Dea et al. (2017) and Rhodes et al. (2017). Our focus on aquatic system impacts results from this and other research, which has identified a substantial set of beneficiaries and attributes associated with these impacts. As a result, these studies offer a rich and diverse set of examples for our analysis.

For our set of case studies, we selected economic valuation studies with the following objectives and considerations in mind:

1. Studies representing a mix of different nonmarket valuation method applications
2. Recent peer-reviewed applications of these methods
3. Studies using reasonably well-defined changes to aquatic systems
4. Studies estimating values for different types of beneficiaries.

Table 1 lists and describes the main features of the selected studies. The valuation methods used in these studies fall into two main categories—revealed preference (RP) and stated preference (SP)—which are described in more detail below, along with additional details about each study as provided in Appendix S1: Table S1.

### Revealed preference studies

Economists often use data on people’s observed choices in real-world settings to infer the value they attach to changes in different ecological attributes. In these studies, the observed choices are assumed to reveal individuals’ preferences for the attributes. Random utility models (RUMs) of recreation demand, hedonic property value models, and bioeconomic models are examples of RP methods. These methods and the case studies that use them are briefly described here.

*Random utility models of recreation demand.*—In a RUM, observed choices of recreational (e.g., fishing) sites are primarily modeled as a function of the ecological (and other) attributes of the sites and the travel cost (or price) of visiting the site. Socioeconomic and other attributes of study population are also often included. The observed site choices and associated travel costs are used to infer the value an individual implicitly attaches to the ecological attributes of interest, controlling for other factors. Two of the selected case studies employ this method.

Lipton and Hicks (1999) estimate a RUM to evaluate the welfare effects of water quality changes to striped bass anglers in the Chesapeake Bay. The commodity valued in this study is expected catch rate. This rate is then used to estimate a RUM of site choice. Expected catch rates are linked back to water quality using other related variables such as historic catch rates and angler characteristics. The estimated models were then applied to evaluate the changes in welfare associated with improvements in water quality.

Montgomery and Needelman (1997) employ similar methods to estimate the benefits of elimination of toxic contamination in freshwater fish and reducing acidification levels in New York lakes and ponds. The beneficiaries are recreational anglers in the state of New York. Values for different measures of water quality (and other lake characteristics such as area, elevation, and shoreline) are estimated using a RUM of fishing site choice.

*Hedonic property value models.*— In these models, the price of a house is modeled as a function of environmental/ecological attributes as well as the attributes of the property and the neighborhood. This method decomposes the price of the house to estimate implicit prices of these different attributes. The one selected study using this approach is Poor et al. (2007), which estimates the value of marginal changes in water quality to residents in the St. Mary’s River watershed in Maryland. The beneficiaries and the commodity being valued that are the focus of this study are thus property owners and measures of water quality, respectively.

*Bioeconomic models.*— These models can be viewed as production function approaches where the production of an economic good is modeled as a function of inputs that include ecological attributes. This economic good is exchanged in a market and yields utility. These models employ a two-step procedure. The first step involves determining the physical effects of changes in biological/ecological attributes on an economic good/activity. In the second step, the impacts of changes in the ecological attributes are valued in terms of changes in the output of the economic good/activity. Note that the ecological attributes are not traded in markets and thus do not have observable prices. Therefore, although market prices for economic goods are often used in bioeconomic models to infer the implicit value of the ecological attributes, these methods are still referred to as non-market techniques.

Macmillan and Ferrier (1994) use a bioeconomic model to estimate the economic benefits of the recovery of the rod and line salmon fishery of Galloway, South West Scotland, due to acid rain abatement. Specifically, it estimates the magnitude of potential marginal economic benefits of alternative abatement levels of sulfur dioxide emissions over the period 1988–2038. The beneficiaries that are the focus of this study are commercial fishermen. The modeling components in the paper (represented in Fig. 1e) are as follows:

1. Changes in alternative deposition scenarios to changes in water quality were modeled using a water chemistry model (Cosby et al. 2001).
2. Changes in water quality were translated to healthy fish population status using a fish population model.
3. Changes in fish population status were linked to changes in catch using estimated regression results from a pre-existing study.
4. Changes in catch were translated to a change in market value of catch using an estimated value of a salmon caught by rod and line and a nonlinear function linking percent change in catch to percent change in market value and an assumed elasticity of value with respect to catch.

### Stated preference studies

In these types of studies, people’s responses to survey questions about hypothetical, but realistic and feasible, scenarios are used as data by economists. One variant of SP methods is contingent valuation (CV). A description of the ecological attributes of interest is provided by the researcher to the survey respondent. Values for changes in ecological attributes are estimated by asking people how much they are willing to pay for a change in the attributes. Thus, these methods provide estimates of the value that people attach to a change in the ecological attribute that is defined by the researcher. For our case studies, we selected the following three SP studies that employ CV methods.

Banzhaf et al. (2006) elicited the total economic value of improvements in the ecological attributes of Adirondack Park lakes and related ecosystems damaged by acidification using a survey of New York residents. The commodity valued in this study was the number of undamaged lakes, and the study potentially includes multiple beneficiaries since it considers the general population.

Lipton (2004) used CV to estimate the benefits of water quality to boaters in Maryland. The survey was conducted among boaters who used their boats 50% or more of the time on the Chesapeake Bay. Boaters could derive utility from cruising, fishing, swimming/skiing/tubing, or other activities. Therefore, the study potentially includes all recreational beneficiaries who value water quality and who secure their access by boat.

Macdonald and Boyle (1997) examined the effect of a sport fish consumption advisory on behavior, consumption, and benefits of open water anglers with fishing licenses in Maine. Using data gathered through a survey, the authors estimated annual net economic values for open water fishing with and without fish advisories about mercury contamination. Each respondent was presented with a randomly assigned dollar amount and asked if he or she would pay that amount over what they actually paid to fish open water with and without advisories. Mercury contamination (identified/represented by fish advisories) is therefore the commodity valued in this study and the beneficiaries are recreational anglers.

## Analysis of Case Studies

In this section, we focus on our first goal, which is to evaluate whether the commodities valued in the case studies are specified in a way that is consistent with a FEGS approach. To do this, we examine whether the ecological attributes valued in each of the case studies satisfy the two criteria identified in *Defining and applying a FEGS approach.* To reiterate, (1) the ecological attributes being valued must be biophysical entities that are directly relevant to beneficiaries, and (2) complete sets of ecological, temporal, and spatial attributes that are specifically relevant to the beneficiary must be included.

Applying the analytical framework described in Fig. 1a, the case studies are also represented in Fig. 1b–h. These figures illustrate where and how the studies make the linkages between policy and human well-being. Components necessary to do an end-to-end policy analysis that are not included in each of the valuation studies are blacked out. Since RP studies utilize data on observed behavior and SP studies utilize data collected from surveys, the implications of departures from the criteria are different for these two types of methods. Thus, we organize our analysis and findings by these two broad methods.

### Analysis of studies using RP methods

*Criterion 1 (definition of FEGS metric).—*Among the RP studies reviewed, all of them include commodity measures that satisfy at least one of the FEGS metric criteria; however, only the Macmillan and Ferrier (1994) study satisfies both. The commodities valued in Lipton and Hicks (1999) include (1) water quality measures such as ambient dissolved oxygen (DO) levels, water temperature, chlorophyll, and salinity and (2) predicted fish catch rates (based on water quality and other factors). The authors note that what anglers care about directly is the expected stock of fish (a FEGS metric) available for catch at a particular location and time. However, in the absence of data on information about stock of fish, water quality measures and their effects on expected catch rates were used as a proxy. To justify this approach, the authors cite prior work that shows a statistically significant correlation between fish stocks and water quality measures such as DO and temperature.

Based on the first criterion, neither the expected catch rates nor the water quality attributes valued in the study fully satisfy the definition of a FEGS metric. Water quality measures such as DO levels and water temperature are purely biophysical, and hence, criterion 1a is satisfied. However, as seen from Table 3, these measures were not identified to be FEGS metrics for recreational anglers either in Ringold et al. (2011, 2009) or by the authors of the study. Rather, they can be interpreted as intermediate metrics in the FEGS framework (see Fig. 1b) because of their role in the ecological production of fish. Therefore, criterion 1b is not satisfied. Expected catch rates do not satisfy criterion 1a because they are not purely biophysical. They involve human inputs such as angler experience, technology, and hours spent on fishing.

Montgomery and Needelman (1997) estimate values using water quality measures and measures of toxicity from fish consumption. According to the authors, stocks of fish can be impaired by pH levels. Consequently, a variable denoting pH threatened (representing pH from 6.0 to 7.0) or pH impaired (representing pH less than 6.0) was included in the study. The fish toxicity measure was derived from fish advisories in problem sites and represented by two levels of warning—eat none of the fish caught, or eat no more than one per month.

The authors note that studies that use catch or stock of fish to value fishing opportunities suffer limitations when used to examine changes in water quality policy. First, using such measures requires the difficult step of quantitatively linking pH to fish stocks or fish catch. Second, they note that toxic contamination will usually impact human health before it affects fish stocks (and consequently catch rates).

Like the DO and temperature variables used in the Lipton and Hicks study, pH is not a FEGS metric for a recreational angler. Although criterion 1a is satisfied (pH is a measurable biophysical attribute), criterion 1b is not satisfied because anglers do not directly care about or observe pH. As shown in Fig. 1c, it is an intermediate attribute.

In contrast, the fish toxicity measure does meet the criteria for being a FEGS metric. First, despite being represented by categorical variables, fish advisories are based on biophysical measures of toxicity of fish. Thus, criterion 1a is satisfied (even though the toxicity of fish was not identified as a FEGS metric in Ringold et al. (2011, 2009)). Second, because the toxicity variables are derived from public fish advisories, they are directly relevant to anglers and require little translation. This implies that criterion 1b is also satisfied.

The measures of water quality valued in Poor et al. (2007) were total suspended solids (TSS) and dissolved inorganic nitrogen (DIN). Both measures are associated with runoff from agricultural and urban lands, but some of the nitrogen is likely to originate from atmospheric deposition to the watershed. Dissolved inorganic nitrogen satisfies criterion 1a since it is purely biophysical. However, it is not directly relevant to residential property owners and can be considered more intermediate in the FEGS framework (Fig. 1d). Thus, it does not satisfy criterion 1b and, as seen from Table 3, is not identified to be a FEGS metric for this category of beneficiaries. Although not a direct measure of clarity such as Secchi depth, clarity is heavily influenced by TSS. We interpret TSS as a purely biophysical measure of clarity, which means criterion 1a is satisfied. If interpreted as a measure of clarity, TSS is an attribute that contributes directly to the utility of residential property owners. However, TSS still requires some translation, and thus, criterion 1b is not satisfied. Brezonik (1978), for example, developed such a translation in the form of a mathematical model relating TSS and color to Secchi disk depth.

The study by Macmillan and Ferrier (1994) applies a fish population model to translate water quality to probability of healthy fish status in six major salmon rivers. As the goal of the study is to estimate the change in the value of the fisheries, the authors use estimates of the probability of healthy fish stock to link water quality to changes in catch rate. We note that the healthy fish status measure is a combination of fish quality and abundance. As discussed before, Ringold et al. (2011, 2009) did not include quality characteristics of fauna as FEGS metrics. However, since the attribute valued is both biophysical and directly relevant to commercial anglers (Fig. 1e), both 1a and 1b are satisfied, and this measure is broadly consistent with the definition of FEGS.

Of the seven studies that we evaluate in this paper, this is the only study that specifically estimates the welfare impacts of altered atmospheric deposition levels and thus conducts an end-to-end policy analysis. As Fig. 1e shows, the study provides explicit links between changes in emissions, intermediate attributes such as pH, alkalinity, and ANC (acid neutralizing capacity), probability of healthy fish status, and catch.

*Criterion 2 (complete and well-defined set of beneficiary-specific biophysical, spatial, and temporal attributes).—*As can be seen from comparing the third and fourth columns of Table 3, none of the four RP studies described above includes all the biophysical attributes deemed to be important in Ringold et al. (2011, 2009). For example, the first row corresponds to the Lipton and Hicks (1999) study that focuses on recreational anglers. The third column provides examples of the attributes that were deemed to be relevant for recreational anglers in Ringold et al. (2011, 2009). The last column lists the attributes valued in the Lipton and Hicks (1999) study.

Temporal and spatial attributes (summarized in Appendix S1: Table S1) are discussed in the papers. For example, Lipton and Hicks (1999) describe the spatial and temporal methods for linking water quality with the recreational site and provide reasonable justification for their methods. Montgomery and Needelman (1997) also describe methods used to spatially and temporally link angler data, site data, and water quality data. Water quality at monitoring station closest to house and for the same time period was used in the study by Poor et al. (2007). The Macmillan and Ferrier (1994) study also provides some information on spatial and temporal attributes. However, the level of detail varies across the studies.

### Analysis of studies using stated preference methods

*Criterion 1 (definition of FEGS).—*Among the SP studies reviewed, all define commodities of direct relevance to beneficiaries (i.e., they satisfy criterion 1b); however, they are mixed in satisfying the other FEGS metric criterion. The ecological attributes valued in Banzhaf et al. (2006) were numbers of healthy vs. unhealthy lakes or lakes of concern (see Fig. 1f; birds and tree species were also described as being affected by acidification, but this is not our focus since we restrict our analysis to aquatic ecosystems). The survey describes lakes of concern as unhealthy lakes where fish and other aquatic life have been reduced or eliminated because of air pollution in the past (Adirondacks Version of Survey, September 2003). The survey asks respondents whether they would pay a certain amount for policies that would improve the unhealthy lakes over a period of 10 yr (using lime to neutralize the excess acidity). Although the variables are biophysical in nature, what is considered a lake of concern may be open to interpretation. The attributes valued in the study may be viewed as labels of FEGS and potentially have an element of subjectivity rather than being objective measurable quantities. However, the survey describes in some detail what an unhealthy lake is in terms of fish and there is an explicit effort to limit subjectivity. Hence, criterion 1a is broadly but not perfectly satisfied. The study used a scientific review and focus groups to help convey scientific information in a meaningful way to respondents. The translation between measurable quantities and directly relevant and understandable measures was made in an effective manner, and criterion 1b is satisfied. The study therefore provides an example of how uncertainties arising from not satisfying criteria 1 may be minimized by using well-designed surveys.

The Lipton (2004) study describes water quality categories (Fig. 1g) associated with health impacts of water. Respondents were presented with an ordinal ranking of water quality on a scale of 1–5 (ranging from poor to excellent) and were asked to rate the water quality. They were asked to choose what their primary concern about water quality was among five different options. These ranged from no concerns, unpleasantness to different levels of health concerns due to toxic chemicals and harmful algal blooms (HABs). The respondents were then asked what their willingness to pay (WTP) would be for a pollution-reduction program to improve the water quality one step from how the respondent ranked it.

The attributes described in this paper represent people’s interpretation about the status of water quality and so the estimated values are not tied to specific biophysical attributes that contribute to boating experience. Criterion 1a is thus not satisfied. However, if water quality rankings were translations of biophysical attributes such as amount of water and water quality characteristics such as chemical contaminants, HABs, and pathogens, criterion 1a would be satisfied. People’s interpretation of the status of water quality is directly relevant and does not require any translation, which implies that criterion 1b is satisfied.

Macdonald and Boyle (1997) value changes in fish advisories (Fig. 1h) to angler beneficiaries. Although not biophysical measures by themselves, advisories are based on measures of toxic contamination. Thus, criterion 1a is satisfied. Advisories can be viewed as expert translations of highly technical measures that make those measures relevant to people, and they therefore satisfy criterion 1b. As a result, though advisories were not a FEGS metric identified in Ringold et al. (2011, 2009), they are broadly consistent with a FEGS approach.

*Criterion 2 (complete and well-defined set of beneficiary-specific biophysical, spatial, and temporal attributes).—*Banzhaf et al. (2006) estimate the value of improvements in acidic lakes to New York residents. The study does not explicitly identify specific beneficiaries of the ecological attributes. The benefit estimates from this study are, as is the common practice with SP analysis, described as total use. It is understood to capture both use and non-use values derived from the ecological attributes. In principle, total use captures the value of all the sets of beneficiaries and the different ways in which they benefit from changing attributes.

Lipton (2004) and Macdonald and Boyle (1997) identify specific beneficiaries in their study. Lipton (2004) conducts a survey of boaters who could potentially undertake cruising, fishing, swimming/skiing/tubing, or other activities. Thus, the relevant beneficiaries could include nature viewers, waders, swimmers, anglers, and boaters. Macdonald and Boyle (1997) focus on recreational anglers. Comparing the third and fourth columns of Table 3, it can be seen that these two studies did not include all the attributes identified to be important to relevant beneficiaries in Ringold et al. (2011, 2009).

All the SP studies include some spatial and temporal information (summarized in Appendix S1: Table S1). However, the level of detail varied across the studies. For example, although the Lipton (2004) study included a comment describing improvement as relatively permanent water quality improvement, it did not explicitly specify whether the water quality improvement will be over a period of time, how long it will last, etc.; Banzhaf et al. (2006), on the other hand, do specify the improvement to occur over a period of 10 yr.

## Implications for Policy Analysis

In this section, we address the second and third goals of the paper, which are to examine (1) the implications for conducting end-to-end policy analysis when commodities valued in the case studies are not consistent with a FEGS approach and (2) whether a FEGS approach can help in designing valuation studies that are conducive to transparent and credible benefit transfers. Table 4 summarizes the main findings of the case study analysis and associated implications. The first column lists the evaluation criteria for modeling. The second and third columns summarize our analysis of RP studies and the associated implications for conducting policy analysis. The last two columns provide a similar summary for the SP studies.

Spatial and temporal attributes are described in all the case studies (both RP and SP). However, the degree of detail varies across the studies and it may be more challenging to conduct benefit transfers using estimates from studies with less detail. Conducting benefit transfers may require more assumptions and therefore introduce more uncertainty. Below, the implications of departures from other FEGS criteria are summarized separately for RP and SP studies.

### Implications for studies using revealed preference methods

*Criterion 1 (definition of FEGS).—*A main potential advantage of the RP case studies for conducting end-to-end policy analysis is the ability to link the policy changes directly to the changes in ecological attributes valued in these studies. These studies generally satisfy criterion 1a because they value changes in measurable biophysical attributes. These changes in the water quality can be linked to loadings using models for watershed chemistry by a policy analyst when applying the estimated results to an end-to-end policy context.

Because they do not focus on end-products, the RP studies (except for Macmillan and Ferrier 1994) do not satisfy criterion 1b. Conducting benefit transfers can still present challenges. For example, consider studies that only use water quality as a proxy for fish stocks. If the relationship between these two measures is not stable across time or space due other varying factors that also influence fish stocks (e.g., presence of predators or prey), then there will be greater uncertainty when transferring RP value estimates based on water quality changes.

*Criteria 2 (complete and well-defined set of beneficiary-specific biophysical, spatial, and temporal attributes).—*Data availability concerns typically limit the ability to include all relevant attributes in RP studies. Consequently, conducting benefit transfers may require the analyst to make assumptions. For example, suppose we were to focus on recreational anglers and consider a policy that changed deposition. This would likely result in a change in not just the water quality (and consequently stock of fish), but also the attributes that contribute to sensory experiences associated with recreational angling (such as visual characteristics and odor). The recreational angling case studies primarily focus on water quality attributes. If water quality can be considered a perfect proxy for all attributes relevant for the angling beneficiary, then simply transferring the value of water quality attributes can capture all the changes. However, if water quality cannot be considered a perfect proxy, not including additional attributes that contribute to sensory experiences (provided the data are readily available) may provide an incomplete range of impacts. Even if the policy in question changes only some of the attributes that are relevant to the beneficiary, transferring values from a study that includes all attributes can help reduce uncertainties. This is because the attributes may be correlated and values associated with each attribute may not be separable from others.

### Implications for studies using stated preference methods

*Criterion 1 (definition of FEGS).—*Some SP studies do not strictly satisfy criterion 1a since the commodity valued is not explicitly tied to an objective biophysical measure. Making explicit policy linkages using the Lipton (2004) study would require assumptions about what thresholds would be used for defining poor water quality vs. good water quality. Since these measures are subjective and open to interpretation, linking these measures to biophysical measures to support end-to-end policy analysis can thus be challenging and result in uncertainties. Explicit efforts to link the subjective measures to biophysical measures as done in Banzhaf et al. (2006) or using measures that are grounded in biophysical measures such as fish advisories (used by Macdonald and Boyle 1997) could considerably help reduce uncertainties.

*Criterion 2 (complete and well-defined set of beneficiary-specific biophysical, spatial, and temporal attributes).—*Similar to the RP studies, there is a potential for obtaining estimates that can better support end-to-end policy analysis if all attributes were considered in future studies. Studies such as Banzhaf et al. (2006) that focus on total use potentially incorporate the full range of attributes and beneficiaries. However, in the Banzhaf et al. (2006) study (and other similar CV studies), the same commodity is described to all beneficiaries. Under the FEGS approach, different beneficiaries may care about different sets of attributes and there may be substantial preference heterogeneity across beneficiaries. For example, a fisherman may not be interested in the health of the entire lake but simply the fish abundance. Although a healthy lake contributes to fish abundance, to a fisherman this is an intermediate attribute. But health of the entire lake may be important for beneficiaries with non-use values. Uncertainties may arise when conducting benefit transfers to other policy contexts since the set of beneficiaries may be different from the set that was relevant for the valuation study.

## DISCUSSION

Conducting an end-to-end policy analysis requires (1) accurate value estimates of changes in ecological commodities that contribute to human well-being and (2) integrating ecological and economic valuation models. However, resource and/or time constraints often necessitate the use of benefit transfer methods. Policy analysis, which requires drawing connections between ecological and valuation models, is complex due to the interdisciplinary nature of the task and lack of data. The assumptions that need to be made to make credible benefit transfers often result in uncertainties. Our premise is that a FEGS approach can help minimize such uncertainties for the following reasons. First, since FEGS metrics are based on biophysical measures that are directly relevant to beneficiaries, the approach allows for links to be easily established among policy, natural sciences (ecological models or measures), and social sciences (economic valuation models). Second, a FEGS approach identifies all the beneficiary-specific biophysical attributes and what the user really cares about, and emphasizes the importance of temporal and spatial attributes. This allows for benefit transfers to be conducted with minimal assumptions and transparency.

To summarize, we find that the selected studies are not always consistent with the FEGS approach, in many cases due to data limitations. As noted by some of the case studies, all necessary data are not usually available. Some studies note that using intermediate attributes (or indicators) of FEGS metrics may be the only alternative available when conducting RP studies, even though not using end-products may result in uncertainties when conducting benefit transfers. Identifying and gathering data on metrics that are directly beneficiary-relevant is an important step that needs further research and collaboration between natural scientists and social scientists.

We also find that in general, inconsistencies with the FEGS approach have different implications for conducting end-to-end policy analysis using RP vs. SP studies. Revealed preference studies often use data on factors that are determinants of end-products (i.e., intermediate indicators) as proxies. If linkages between F—ecological attributes being valued—and V—human wellbeing—are difficult to establish, the analyst may need to use strong assumptions when conducting benefit transfers and this may introduce uncertainties. However, it is important to note that using proxies does not necessarily limit the ability to provide reliable valuation estimates. For example, the RP case studies estimate values for different chemical/physical characteristics of water, which are used as surrogates for the end-products that angler or homeowners really care about. As long as these water quality measures are highly correlated with the relevant FEGS metrics (such as stock of fish), then fairly accurate value estimates may be obtained in an RP context. That is, even though individuals do not directly perceive or understand these water quality measures, they are nonetheless good proxies for the relevant end-products.

However, for SP studies to provide policy-relevant estimates, it is essential for the attributes being valued to be directly relevant and readily comprehensible since people are asked to state their WTP for changes in this commodity. For example, anglers, a beneficiary who directly value fish and site appeal, may find it difficult to meaningfully state their WTP for pH or measures of nutrients. To provide a meaningful statement, they would have to translate changes in these chemical measures into changes in the fish stocks that they value. Any uncertainty in their capacity to do this would be embodied in uncertainty in their WTP. If biophysical measures are employed to provide the linkage between P—policy—and F—ecological attributes being valued—it may, depending on the variable, be necessary for a researcher to provide the linkage between F and V—human well-being. For example, fish advisories could be viewed as a translation of a biophysical measure of toxicity of fish into categories that resonate with people such as safe to eat fish or not. However, translating biophysical measures that can be linked to policy changes into measures that are readily understandable by survey respondents is challenging. Use of focus groups (Weber and Ringold 2012, 2015) to better understand what beneficiaries care about and can comprehend and explicitly using this information when designing surveys can help reduce uncertainties in conducting benefit transfers. It is also a useful way to understand how beneficiary preferences vary, for example, across ecosystems or regions, demography, or social context.

The need for any level of aggregation or disaggregation of beneficiaries and the commodities which they directly value is an important and long-standing research issue. Although it is possible to identify many beneficiary-commodity combinations that are potentially affected by, for example, changes in atmospheric deposition (O’Dea et al. 2017, Rhodes et al. 2017), it is often challenging to separate out the specific beneficiary-commodity connections that are captured in or excluded from certain economic valuation estimates. This disaggregation issue arises in both our SP and RP case studies. In the SP context (Banzhaf et al. 2006), studies typically estimate total use benefits which captures, in principle, all the sets of beneficiaries and the different ways in which they benefit from the attributes. However, care must be taken and uncertainties must be acknowledged when using such studies to transfer benefits to contexts where the relevant set of beneficiaries is different. Studies that value attributes relevant to specific beneficiaries require fewer assumptions when applied in benefit transfers.

Continued efforts to disaggregate beneficiaries by ways in which they derive value from FEGS metrics would be useful in further research. To determine the value of the attributes, it is important to identify not just broad categories of beneficiaries but also the ways in which the beneficiary uses the attribute. For example, the Macdonald and Boyle (1997) RP study emphasizes that although advisories are directly relevant to anglers, the extent of relevance varies with different types of anglers. The study explores the impacts of changes in fish advisories on fish populations who are at different levels of risk. Female anglers of reproductive age who consume their catch face relatively high risks and are therefore more likely to care about advisories than those who do not consume the fish they catch.

It is worth noting a few important caveats for applying the FEGS approach. First, our assumption is that the initial research conducted by Ringold et al. (2011, 2009) identified all beneficiary-relevant attributes. However, this analysis illustrates that quality characteristics of fish, while broadly consistent with the definition of a FEGS metric, was not one of the relevant attributes identified. Consequently, the list of attributes that beneficiaries care about provided by Ringold et al. (2011, 2009) should be viewed as a starting point, and reviewing valuation studies can help expand this list. Second (as noted by Poor et al. 2007), using intermediate attributes may allow for capture of a broader range of attributes relevant to beneficiaries. For example, in the absence of data on all relevant water quality attributes and aquatic life, using intermediate attributes such as submerged aquatic vegetation or nutrients may be more appropriate than using simply fish stocks. This is because these intermediate attributes may be an indicator for water providing aesthetic benefits, fish providing recreational benefits, turtles providing aesthetic benefits, etc. The third caveat is raised in some of the case studies using RP methods—if final attributes are valued, there is more of a burden on biophysical or chemical modeling. For example, suppose economic models estimate values for changes in stocks of fish. In order to link changes in fish stocks to atmospheric policies that result in water quality changes, it would be necessary to use models that would translate changes in water quality to changes in fish stocks. There may be associated uncertainties in this type of modeling as well, and thus, there is a potential tradeoff between using final and intermediate attributes in such contexts.

Keeping these caveats in mind, we nonetheless expect that using FEGS metrics to identify the complete set of beneficiary-specific attributes can provide economists with a useful starting point for considering how the commodity can be defined and specified in the valuation study. Even if data limitations may prevent economists from including all relevant attributes, a detailed description of the biophysical, temporal, and spatial attributes can still support end-to-end policy analysis. Identifying all the key attributes of the commodity valued can greatly help in determining whether the context in which the original study was conducted matches with the policy context. This may, for example, help in both determining the extent of uncertainty and developing error ranges.

## Supplementary Material

Sup 1

## Figures and Tables

**Table 1.** Summary of case studies.

Study	Valuation method	Biophysical measure (S)	Water resource	Study area	Study population/beneficiaries	Source of impairment
1. Lipton and Hicks (1999)	RUM travel cost (RP)	Dissolved oxygen and striped bass catch rates	Estuary	Chesapeake Bay	Recreational anglers	Nutrient loads including from deposition
2. Montgomery and Needelman (1997)	RUM travel cost (RP)	pH ranges indicating impairment levels	Lakes	New York	Recreational anglers	Acid deposition
3. Poor et al. (2007)	Hedonic property value (RP)	Dissolved inorganic nitrogen and TSS	Estuary	Chesapeake Bay	Property owners	Nutrient (and sediment) loads including from deposition
4. Macmillan and Ferrier (1994)	Bioeconomic (RP)	pH and salmon population changes	Catchments	SW Scotland	Commercial fishers	Acid deposition
5. Banzhaf et al. (2006)	CV method (SP)	Percent of impaired lakes	Lakes	Adirondacks	General population	Acid deposition
6. Lipton (2004)	CV method (SP)	5-level water quality indicator	Estuary	Chesapeake Bay	Boaters	Nutrient (and sediment) loads including from deposition
7. Macdonald and Boyle (1997)	CV method (SP)	Mercury fish advisories	Open waters	Maine	Recreational anglers	Mercury deposition

*Notes:* CV, contingent valuation; RP, revealed preference; RUM, random utility model; SP, stated preference; TSS, total suspended solids.

**Table 2.** Key FEGS features for monitoring and evaluation criteria for modeling welfare impacts.

Key FEGS features important for ecological monitoring	Evaluation criteria for modeling welfare impacts
1. The ecological commodity being considered must satisfy the definition of a FEGS metric: “Biophysical features, quantities and qualities requiring little further translation to make clear their relevance to beneficiaries” (Ringold et al. 2009)	a. Ecological attributes being valued must be biophysical measurable entities: This allows for P-to-F linkage to be established b. Ecological attributes being valued require little translation to make clear their relevance to beneficiaries: This allows for F-to-V linkage to be established; using metrics that are directly relevant provides more accurate benefit estimates that are easy to transfer to other policy contexts
2. Different beneficiaries care about or benefit from different sets of biophysical, spatial, and temporal attributes of the ecosystems: People benefit from ecosystems in diverse ways and each of these ways may require differing representations of the ecosystem.† Each of these ways in which people benefit from ecosystems typically reflects multiple biophysical attributes. Further spatial and temporal attributes are important from a measurement perspective since they are important for defining indicators (Jackson et al. 2000)	a. Complete sets of ecological attributes that are specifically relevant to the beneficiary must be fully represented: Correct and complete representation of relevant ecological attributes is essential for conducting accurate and transparent benefit transfers b. Temporal and spatial attributes of the valued attributes must be well defined: Values of biophysical attributes are typically location and time specific, so including such attributes can help accurate and transparent benefit transfers

*Notes:* FEGS, Final Ecosystem Goods and Services; F, ecological attributes being valued; P, policy; V, human well-being.

† For example, in a stream, a recreational angler derives value from one representation of fish and the appeal of the surroundings, a commercial fisher derives value from a different representation of those same fish and the place in which they are caught, and a farmer derives value from the quantity of water suitable for crop irrigation.

**Table 3.** Evaluation of second criterion: comparison of beneficiary-specific attributes identified in Ringold et al. (2011, 2009) and ecological commodity valued in case studies.

Study	Study population/beneficiaries	Beneficiary-specific attributes	Ecological commodity valued in case study
1. Lipton and Hicks (1999)	Recreational anglers	Site characteristics such as substrate	
		Water quality characteristics such as chemical contaminants, HABs, and pathogens	Oxygen level; temperature
		Stock of fish	Expected fish catch
		Attributes that contribute to sensory experience	
2. Montgomery and Needelman (1997)	Recreational anglers	Site characteristics such as substrate	
		Water quality characteristics such as chemical contaminants, HABs, and pathogens	pH ranges indicating impairment levels; fish advisories
		Stock of fish	
		Attributes that contribute to sensory experience	
3. Poor et al. (2007)	Residential property owners	Site characteristics such as amount of water	
		Water quality characteristics such as clarity, chemical contaminants, HABs, and pathogens	TSS (determinant of clarity) Dissolved inorganic nitrogen
		Stocks of various flora and fauna§	
		Attributes that contribute to sensory experience	
4. Macmillan and Ferrier (1994)	Commercial fishers	Site characteristics such as amount of water	
		Water quality characteristics such as chemical contaminants, HABs, and pathogens	
		Stock of fish	Quality of fish and stock of fish
5. Banzhaf et al. (2006)	General population	Site characteristics such as amount of water and substrate	Percent of impaired lakes
		Water quality characteristics such as temperature, clarity, oxygen level, pH, nutrients, conductivity/salinity, chemical contaminants, pathogens, and HABs	
		Stocks of various flora and fauna	
		Attributes that contribute to sensory experience	
6. Lipton (2004)	Boaters	Site characteristics such as amount of water and substrate	
		Water quality characteristics such as chemical contaminants, HABs, and pathogens	5-level water quality indicator representing perceptions about chemical contaminants, pathogens, and HABs
		Stocks of various flora and fauna	
		Attributes that contribute to sensory experience	
7. Macdonald and Boyle (1997)	Recreational anglers	Site characteristics such as substrate	
		Water quality characteristics such as chemical contaminants, HABs, and pathogens	Mercury fish advisories (translation of measures of chemical contaminants)
		Stock of fish	
		Attributes that contribute to sensory experience	

*Notes:* HABs, harmful algal blooms; TSS, total suspended solids. The attributes were identified to be relevant to the specific beneficiary listed in the second column. Final Ecosystem Goods and Services attributes that contribute to sensory appeal of recreational anglers include visual appearance, odor, taste, sound, and touch. Flora and fauna include stocks of invertebrates, fish, wildlife, and vegetation; biotic integrity; and genetic diversity. Boaters could potentially undertake cruising, fishing, swimming/skiing/tubing, or other activities. Thus, the relevant beneficiaries could include nature viewers, waders, swimmers, anglers, and boaters.

**Table 4.** Summary of analyses and implications for revealed preference (RP) and stated preference (SP) studies.

	RP		SP	
Evaluation criteria	Evaluation	Implications	Evaluation	Implications
1a. Ecological attributes being valued must be biophysical measurable entities	All the case studies satisfy	Potentially easy to link ecological models to the valuation models and conduct benefit transfers to other contexts	One case study does not satisfy criteria since it uses subjective measures that are not directly tied to biophysical attributes. Two case studies satisfy criteria—one uses a categorical representation of biophysical metrics, while another explicitly attempts to link the subjective measures to measurable biophysical attributes	Potential uncertainties in conducting benefit transfers when using the study which does not satisfy the criteria, since strong assumptions are needed to link objective biophysical measures to the subjective measures. Benefit transfers using the other two studies require minimal assumptions
1b. Ecological attributes being valued require little translation to make clear their relevance to beneficiaries	Some attributes in studies do not satisfy (intermediate attributes used as proxies for directly relevant measures)	Potential uncertainties in conducting benefit transfers to other policy contexts	All case studies satisfy	Do not expect major uncertainties in conducting end-to-end policy analysis
2a. All ecological attributes that are specifically relevant to the beneficiary must be included	None of the case studies satisfy this criterion† (studies do not include all the attributes that were determined to be directly relevant to the beneficiary in Ringold et al. (2011, 2009))	Potential for less uncertainty and more transparency in conducting benefit transfers if FEGS metrics are used	Case studies do not satisfy this criterion. Two studies do not include all the attributes that were determined to be directly relevant to the beneficiary in Ringold et al. (2011, 2009). The study adopting total use potentially represents all attributes, but they are not specifically relevant to different beneficiaries	Potential for less uncertainty and more transparency in conducting benefit transfers if FEGS metrics are used
2b. Temporal and spatial attributes of the valued attributes must be well defined	All case studies include temporal and spatial attributes although the degree of detail varies across the studies	Higher uncertainties when conducting benefit transfers to different spatial and temporal contexts for studies with less detail	All case studies include temporal and spatial attributes although the degree of detail varies across the studies	Higher uncertainties when conducting benefit transfers to different spatial and temporal contexts for studies with less detail

*Notes:* FEGS, Final Ecosystem Goods and Services. Four RP case studies and three SP case studies were part of this analysis.

† The attributes identified in Ringold et al. (2011, 2009) were used as benchmarks for evaluating these criteria. The underlying assumption here is that these two studies have correctly identified all attributes specifically relevant to the beneficiary.
