# Supplementary material for: Using a final ecosystem goods and services approach to support policy analysis
Source: Ecosphere. Author manuscript; Available in PMC 2019 Sep 13. (PMC6650162; doi:10.1002/ecs2.2382)
Supplement: Sup 1 [file NIHMS1506711-supplement-Sup_1.pdf]

Ecosphere

Using a final ecosystem goods and services approach to support policy analysis

Paramita Sinha, Paul Ringold, George Van Houtven, and Alan Krupnick

Appendix S1

Table S1. Summary of Key Features of Case Studies

	**Valuation Method**	**Change in Policy/ Stressor**	**Beneficiary**	**Commodity Valued**	**Temporal Attributes**	**Spatial Attributes**
**Lipton and Hicks (1999)**	Random Utility Model (RUM) Travel Cost	Welfare effects of hypothetical policy to improve water quality to threshold levels.	Recreational anglers targeting striped bass in the Chesapeake Bay area	Expected catch rate and water quality measures obtained from Monitoring Data (ambient dissolved oxygen levels and water temperature at bottom and surface) Bottom dissolved oxygen)	"For temporal matching, the period of water quality sampling that was just prior to the fishing occasion was matched with the fisher. Water quality prior to the fishing date was used rather than the sample closest to the date of fishing because water quality is treated as impacting fishing expectations, not the realization of catch."	Data from interviews of fishers at the 407 intercept sites that are located in the Chesapeake Bay matched with matched with the nearest water quality station that was within 5 km.
**Montgomery and Needelman (1997)**	Travel Cost	Eliminate toxic contamination in lakes, Change all lake pH to > 7 and both of these	New York resident anglers' day trips	Some toxic Species, Extra warning: Eat None; pH Classes (impaired < 6—5% of lakes, threatened 6 to 7—6% of lakes,	1988 for one source	Lakes larger than 2.6 hectares near "mapped town"; whole lakes or segments of large lakes

	Valuation Method	Change in Policy/ Stressor	Beneficiary	Commodity Valued	Temporal Attributes	Spatial Attributes
				suitable for trout > 7		
**Poor et al (2007)**	Hedonic property value	Not Defined. Marginal Willingness to Pay for a one-unit change (mg/L) in the dissolved inorganic nitrogen and a one unit (mg/L) increase in total suspended solids (TSS)	Residential property owners within the St. Mary's River watershed	Annual averages for total suspended solids (TSS) and dissolved inorganic nitrogen (DIN) between June 1999 and May 31 2003	Annual average values of pollutants and housing prices used between June 1999 and May 31 2003	Water quality at monitoring station closest to house
**MacMillan and Ferrier (1994)**	Bioeconomic	Three deposition scenarios: (1) Constant 1988 SO_2 emissions levels (2) 60% reduction from 1990 levels by 2003 and (3) a 90% reduction from 1980 levels by 2008	commercial fisherman	estimated five-year average rod &line salmon catch in a particular year	annual values from 1988 to 2038	Salmon fishery in Galloway, South West Scotland
**Banzhaf et al (2006)**	WTP Contingent Valuation Survey	Not Defined. Analysis conducted in terms of seeking responses to changes in FEGS without a specific link to a specific policy change.	Households in New York State	"Injury" to defined numbers of lakes, area of 3 forest types, populations of < 5 bird species	Ten Year Extent	Adirondack Park
**Lipton (2004)**	Contingent Valuation Method	(Hypothetical) Pollution-reduction program to improve the water quality one step from how you ranked it on [question on	Boaters who used their boats 50% or more of the time on Chesapeake Bay used for survey but estimates extrapolated to statewide	NA (implicit in quality perception categories)	Annual WTP estimates	Chesapeake Bay (extrapolated to MD)

	Valuation Method	Change in Policy/ Stressor	Beneficiary	Commodity Valued	Temporal Attributes	Spatial Attributes
		how you rate water quality]	figures (since water quality in other water bodies may also improve)			
**MacDonald and Boyle (1997)**	Contingent Valuation Method	Estimating annual net economic values for open water fishing **with and without fish advisory**	Maine open-water anglers	NA	Annual values of WTP, questionnaires mailed in October 1994, right after the open-water fishing season	Open-water fishing occurs on waters within Maine Above the tidal zone of rivers and streams when the waters are at least partially free of ice
